# Can Existing Knowledge on Eating Behaviors and Obesity Support People with Cystic Fibrosis Who Are Nutritionally Compromised?

**DOI:** 10.3389/fpsyg.2016.01477

**Published:** 2016-09-27

**Authors:** Michail Mantzios, Helen Egan, Carolyn Patchell

**Affiliations:** ^1^Department of Psychology, Birmingham City UniversityBirmingham, UK; ^2^Birmingham Children's Hospital, NHS Foundation TrustBirmingham, UK

**Keywords:** eating behaviors, obesity, cystic fibrosis, undernourishment

Nutritional status is a key predictor of health outcomes and survival for individuals with Cystic Fibrosis (CF). A main concern is the maintenance of a healthy body weight by eating a high-energy and high-fat diet (Abbott et al., 2008). This diet is accompanied for most patients with pancreatic enzyme replacement therapy, and for some patients with fat soluble vitamins, oral supplements, and enteral tube feeding which further supports the high nutritional requirements (Powers et al., 2002). Nevertheless, inadequate intake in CF remains a major problem within CF populations due to a number of complex reasons including physiological factors such as anorexia or poor appetite (Durie and Pencharz, 1989), early satiety and abdominal pain (Pumariega et al., 1986). Social and psychological factors for inadequate intake in CF include feeling under pressure to eat (Abbott et al., 2008), and being afraid of choking (Murphy and Wootton, 1998). The latest Cystic Fibrosis Foundation Registry Report (2004) indicates that approximately 31% showed symptomatology consistent with malnutrition.

This paper suggests possibilities for developing research further and enhancing behavioral interventions for malnourished individuals with CF. With a paucity of research on the experiences of eating in adults with CF, known information around eating in other populations should be explored. Findings in obesity research may provide useful suggestions for environmental and behavioral interventions with malnourished individuals until a healthy weight status is achieved.

## What do we know from research on obesity?

Clinicians, researchers and health professionals have been struggling for decades to provide workable solutions to the growing obesity problem. While extensive literature has not provided long term solutions to the complex issues of obesity, it offers some findings as to which behaviors and environmental contexts contribute to the increase of consumption, essentially showing how easy it can be to overeat. Four factors were identified from the literature which may be of use in addition to already existing nutritional counseling in CF. People with CF already have a heavy medical regimen which does not easily allow for additional interventions in their daily treatment routines. Being able to make small changes in the environment for the purpose of increasing calorific intake with minimal self-regulation and effort would be a positive outcome for both patients and health professionals. This paper proposes the potential usefulness for people with CF of four research findings from obesity with regards to (a) food portion size, (b) food reachability, (c) food visibility, and (d) unconscious or automatic eating; all of which have been indicated to contribute toward the increase in calorie intake (e.g., Duyff et al., 2015; Roe et al., 2016).

Research has shown that having larger portion sizes available increases calorie intake. For example, having a larger portion at a restaurant or being given a larger bag of potato crisps can increase the calorie intake by between 50 and 150% (Diliberti et al., 2004; Rolls et al., 2004). Second, convenience has been shown to increase calorie intake. Painter and Wansink (2002) observed that office workers who had chocolate sweets on their desk (i.e., at arm's length) consume 5–6 times more than workers whose dish was placed 2 m away. Third, in a similar experiment simply switching between jars that are non-transparent to transparent ones tripled the consumption of chocolate by office workers (Wansink et al., 2006); high visibility of food is therefore another way in which calorie intake is increased. This type of research and the multiple replications demonstrate how environmental cues contribute to problematic overconsumption and obesity, but may be useful findings to aid individuals with CF to increase their calorie intake.

The fourth recommendation is that eating can be transformed into a more automatic behavior, whereby engaging in secondary activities while eating (e.g., reading the newspaper or watching television) increases the likelihood of extending the mealtime, and in effect, increasing situational intake (Tuomisto et al., 1998; Oldham-Cooper, 2011; Boulos et al., 2012; Deng and Srinivasan, 2013). This suggestion to multitask entails engaging in another everyday behavior and this behavioral alignment shifts attention away from the food and alters the eating setting into an environment with distracting stimuli, which may increase consumption. These examples show how the environment and behaviors can significantly contribute to patterns of overeating for some people without them being aware. Environmental factors and everyday pleasurable behaviors are more amenable to change than are health behaviors that need alteration, and this may prove helpful for people who are struggling to attain or sustain optimal nutritional status.

At present however, there is not enough robust empirical research to establish whether these environmental changes would result in an increased calorie intake for people with CF. In clinical terms, the urgency of regaining weight for each patient should guide how and when information on encouraging “overeating” should be used. Research in obesity demonstrates that over-consumption of calories is often made up of foods that are of low nutritional value. While the increased eating of such foods would possibly lead to weight gain and associated improvements in lung function for people with CF, there is also a need to consider how the overall nutritional status of each person can be improved.

The appropriateness of the above suggestions depends in part on the severity of the current individual nutritional status. Additionally, deterioration of respiratory function, inflammation or infection, lack of appetite and abdominal pain must also be considered on an individual basis, while taking into account the long-term effects of these interventions (Powers et al., 2002; Nash et al., 2014). For example, on a short-term basis and at the discretion of the CF multidisciplinary team, individuals who are at high risk may benefit from larger availability of energy dense foods and these foods may be suggested for consumption with the recommendations suggested. For the long-term, focusing on a balance of nutrition and including healthier energy dense foods such as those containing mono-unsaturated fats and oils, oily fish and a range of fruits, vegetables and nuts, may well be implemented with the use of the tools presented above. Alternative implementations may relate to the medication that is required for the majority of individuals with CF. For example, the use of pancreatic enzymes to assist the digestion and the absorption in the gut is a nutritional requirement. Drinking plenty of fluids when taking pancreatic enzymes is one of the directions given to individuals with CF, and fluids can be in larger bottles or glasses and made more visible, reachable and automatic.

It should be noted that if and when a person reaches a healthy body mass index (BMI), how eating settings may be adjusted to maintain a balanced and healthy calorie-dense diet and may entail readjusting environmental changes. For example, putting the bigger plates and glasses in the storage, and replacing them with the old ones does not entail any behavioral change throughout the process of adjusting the environment. Additionally, automatic eating, and for example, eating in front of the television, may require further counseling to readjust to a more desirable eating attitude and behavior. In fact, other nutritional counseling and interventions may become more relevant with the primary focus on mindful, self-regulated and self-controlled eating (see Egan and Mantzios, 2016). These issues warrant discussion, and further investigation before embedding in current nutritional practice.

## What we do not know from CF research

At present, relatively little information exists in regards to eating behaviors of adults with CF; the research that does exist, focuses on detection of disordered eating (Randlesome et al., 2013). The focus on problematic eating consistently notes that individuals with CF display a preoccupation with food, exercise, and weight (Egan and Mantzios, 2016). The question here is whether this constitutes clinical disordered eating, or, whether it is highly attentive, self-regulated, and mindful eating (see Egan and Mantzios, 2016).

There is little known about the eating behaviors and attitudes of adults with CF and understanding more about this could inform present and future counseling and interventions. For example, research could use existing scales to investigate in people with CF the prevalence of external eating (i.e., eating more in response to external food cues such as the sight of food), and/or emotional eating (i.e., eating in response to emotions and feelings), which are found to increase calorie intake (Van Strien et al., 1986, 1995, 2012). There is a need to understand more about how eating is experienced by adults with CF, both for those who may have to work at maintaining optimal nutritional intake and those who do not. For clinicians, using scales that measure specific cues to eat would be helpful for tailoring counseling interventions particular to each individual with CF. Both qualitative and quantitative research is needed in this area to develop and support interventions that are both individualized and cost-effective.

To enable researchers and clinicians to develop deliverable, effective outcomes for this population, research could usefully include qualitative research methodology with clinicians who counsel on nutrition in adult CF, particularly dietitians and nurses. It is possible that issues discussed here are familiar to practitioners and the recommendations proposed already exist in practice, but may not have been formally evaluated and are absent from the academic literature. Therefore, the publication of non-significant results relevant to eating behaviors and cystic fibrosis could further assist and inform future counseling and interventions, merely because there may have been attempts at utilizing these suggestions for increasing calorific intake with limited success.

The potential benefits of the present research are evident. First, specific behavioral guidance and environmental changes through nutritional counseling may increase calorie intake. Second, results may provide further empirical support to information provided to patients and carers through the Cystic Fibrosis Trust (CFT, 2010). Third, the suggested counseling and interventions are easily implemented and followed because they require less motivation and effort (e.g., watching an interesting or emotional movie while eating) than behavioral interventions. The four areas of potential guidance explored in this short communication (Food portion size, food reachability, food visibility, and unconscious/automatic eating) neither provide a complete solution to tackle obesity, nor do they provide a complete solution to inadequate intake for people with CF. They offer suggestions for how environmental and behavioral interventions may assist some people in regaining weight, and additional resources for nutritionists to incorporate into existing practice (such as motivational interviewing) when counseling people with CF.

The focus of the proposed interventions suggests alternative methods for weight gain to previously reported pediatric interventions, which mainly focused on reward-based behavioral principles. Stark et al. (1996) investigated such an intervention that was parent/child oriented to assist calorie intake and found the intervention to significantly assist in increasing food intake compared to a waiting list group. Within the intervention, children were told that they could earn a star on their charts for each meal that was recorded. Parents were also coached that television viewing was a privilege to withhold until the child eats the meal (see also Stark et al., 1990, 1993, 1994). Although there is more conscious engagement within a reward-based system of food consumption, the attentional focus of the child might be on the actual reward, and not the food that is consumed. The proposed ideas of the current paper might go side-by-side with previous interventions, and fits within the latest Consensus Report (Borowitz et al., 2002) and the suggested counseling to maximize energy intake. For children, those tools may be used and incorporated to the already existing interventions of parental guidance, with the flexibility of ceasing the effect of the environment once the child has reached a healthy weight. The reward-based interventions are not without limitations; Birch (1999) suggests that the use of such interventions with children may, in the long run, decrease the liking of the contingent activity resulting in a reduction in the quality of the child's diet. They are also limited by age, for an adolescent or adult population the reward based system becomes less effective as parental control is reduced and rewards are more difficult to negotiate.

Furthermore, the environmental changes suggested in this paper may be an additional or alternative tool for parents in adolescent years—where psychosocial and peer-pressures may dictate a thinner body, especially in females (e.g., Lai et al., 1998). The responsibilities toward self-care involved in adult living for people with CF to adhere to a complex medical regimen, and to achieve optimal nutritional intake and body weight, demand significant cognitive resources. Those tools may serve as an element of increasing intake without adding the stress and frustration of consciously and forcefully having to eat more (because it occurs automatically).

In an evaluation of the effectiveness of nutritional interventions on weight gain, including oral supplementation, enteral nutrition, parenteral nutrition, all were evaluated to be equally successful (Jelalian et al., 1998). However, enteral and/or parenteral nutrition, have been associated with lower quality of life, isolation, stigmatization and possible medical complications (see Candusso et al., 2002; Brotherton and Judd, 2007). Therefore, the need to improve behavioral interventions and adherence to oral supplementation appears to be of primary importance.

Psychological theory attempting to explain why people fail to inhibit their behavior (e.g., Muraven and Baumeister, 2000) suggests that self-regulation is depleted by the overuse of cognitive resources, which is evident when people try to regulate their weight (Vohs and Heatherton, 2000), similar to a muscle that is overworked and gets tired and weak; that is, self-regulatory depletion. This way, people are tempted to indulge (or not) in foods that are inappropriate to their weight regulation goals, and may well lack in self-regulating other health behaviors (such as abstaining from alcohol) or predispose people with CF to a decline in medical adherence. Overall, the minimal cognitive effort that is required by individuals with CF, and the preservation of self-regulatory resources (Muraven and Baumeister, 2000; Chow et al., 2015; Schmeichel et al., 2015) is of additional psychological value. The ability to assist undernourished individuals with CF before the use of invasive medical procedures could save the individual from illness-related stigma and distress (Pakhale et al., 2015), and possibly preserve self-regulatory strength that is much needed in a self-regulatory demanding condition such as CF.

Concerns have been raised about the rising number of overweight and obese adults with CF, and this constitutes a separate, but related topic for further investigation. Evidence to date clearly indicates that low BMI presents a far greater immediate risk to health than being overweight or obese. For overweight and obese adults with CF, the solution may be the exact opposite to mindless eating, and may entail another line of research and advice that relates to mindfulness and self-regulation (see Egan and Mantzios, 2016). The proposed directions of exploratory research would provide useful information for weight regulation for both undernourished and overweight or obese adults with CF.

## Author contributions

MM and HE discussed, conceptualized and wrote the paper together. The manuscript was edited and exchanged multiple times between the authors to reach the final version. CP offered advice and clinical knowledge of applied practice in CF nutrition.

### Conflict of interest statement

The authors declare that the research was conducted in the absence of any commercial or financial relationships that could be construed as a potential conflict of interest.
